# *Nanosynbacter lyticus* gen. nov., sp. nov., an ultrasmall, obligate epibiont bacterium and the proposal of *Nanosynbacteraceae* fam. nov., *Nanosynbacterales* ord. nov. and *Nanosynbacteria* class nov.

**DOI:** 10.1099/ijsem.0.007162

**Published:** 2026-05

**Authors:** Erik L Hendrickson, Batbileg Bor, Xuesong He, Jeffrey S. McLean

**Affiliations:** 1 Department of Periodontics, University of Washington, Seattle, WA, 98195, USA; 2 Department of Microbiology, ADA Forsyth Institute, Somerville, MA 02143, USA; 3 Department of Oral Health Sciences, University of Washington, Seattle, WA, 98195, USA; 4 Department of Microbiology, University of Washington, Seattle, WA, 98195, USA

**Keywords:** candidate phyla radiation (CPR), epibiont, *Minisyncoccota*, *Nanosynbacter*, *Patescibacteria*, *Saccharibacteria*, Nanosynbacteria

## Abstract

The recently validly published phylum *Minisyncoccota* (previously *Ca*. Patescibacteriota or candidate phyla radiation) had long been an enigma, with members having remained uncultivated for several decades since the first SSU ribosomal sequence was recovered. We reported on the first isolated strain of *Minisyncoccota, Nanosynbacter lyticus* type strain TM7x^T^ (ATCC TSD-290^T^), in 2015, using directed cultivation from human oral samples. Strain TM7x^T^ had an extremely reduced genome (705 CDS), ultra-small cell size (200–300 nm), and replicated as an obligate epibiont on the surface of another commonly found oral species: *Pauljensenia odontolytica_C* strain XH001 (host bacterium). TM7x^T^ also exhibited what has been defined as a parasitic phase, where it disrupts the membrane of the bacterial host cell, resulting in host bacteria lysis. However, free-floating, host-free TM7x^T^ cells remain viable and can re-infect fresh host bacterial cells when available. This discovery and coculture marked the first concrete evidence of how these ultrasmall organisms within the *Minisyncoccota* survive and persist despite their reduced genomes missing *de novo* biosynthesis of many essential compounds, including all amino acids and vitamins. This model dual species system has now allowed a deeper understanding of the TM7x–host bacterium interaction and dynamics, with over 15 publications reporting on various aspects of this strain to date. We propose the name *Nanosynbacter lyticus* gen. nov. sp. nov. for strain TM7x^T^. We also propose *Nanosynbacteraceae* fam. nov., *Nanosynbacterales* ord. nov. and *Nanosynbacteria* class nov. to accommodate the genus *Nanosynbacter*.

## INTRODUCTION

The *Candidatus* phylum, *Ca*. Patescibacteria [or the synonym candidate phyla radiation (CPR)], was first described as a distinct phylogenetic group by Brown *et al.* in 2015. The validly published name for this phylum is *Minisyncoccota* [1]. The phylum is broad, by some estimates comprising over 15% of the domain *Bacillati* [2]. Members are found in extremely diverse natural habitats, including soil, seawater, underground water, rhizospheres, deep-sea sediments, wastewater treatment plants, hot springs, termite guts and mammals [3-8]. Mammalian association includes the human microbiome, and 16S rRNA gene amplicon sequencing has found *Minisyncoccota* in various body sites, including the oral cavity, gastrointestinal tract, skin and the female genital tract [3, 9-12]. The phylum is noted for reduced genomes lacking many essential biosynthesis pathways, such as amino acids, vitamins and cell wall precursors [2, 13, 14]. Most interestingly, members of this phylum are generally believed to live as obligate epibionts on host bacteria, a relationship often referred to as episymbiotic [15, 16]. However, there are suggestions that some species may be free living [17].

The ultra-small cell size, reduced genome and episymbiotic lifestyle of these organisms were discovered by He *et al.* in 2014 when they established the first cultured member of this phylum, *Ca. Nanosynbacter lyticus* type strain TM7x^T^, several months before the CPR was first described [18]. TM7x^T^ was cocultivated with its host bacteria, *Pauljensenia odontolytica_C* strain XH001 (RefSeq GCF_001462375.1 and *Actinomyces odontolyticus* XH001) [19], showing TM7x^T^ to be an obligate epibiont. Isolation revealed the ultra-small cell size (200–300 nm) of the organism. The complete TM7x^T^ genome (CP007496.1, RefSeq GCF_000803625.1) was deposited on 18 December 2014 and was shown to be highly reduced. The Genbank assembly predicted 754 genes with 701 encoding proteins. Absent were genes required for *de novo* biosynthesis of essential amino acids, nucleotides and cofactors, which made this strain an obligate epibiont and explained the previous failed attempts in isolating phylum members as pure monocultures.

The cultivation and nomenclature of this species are part of a complex history. The first 16S rRNA gene sequences designated TM7 were detected from a peat bog sample (TM stands for ‘Torf, mittlere Schicht’, German for a middle layer of peat) in northern Germany in the mid-1990s by using a culture-independent molecular approach [20] along with highly divergent sequences from a number of additional candidate *Bacillati* such as TM6 (*Ca*. Dependentiae) [21]. TM7 was later proposed as a distinct *Bacillati* phylum based on partial 16S rDNA sequences [4, 22] and designated ‘candidate division TM7’ or synonym candidate phylum TM7. In 2013, four complete TM7 genomes were assembled through differential coverage binning sampled from an activated sludge bioreactor [23]. ‘Candidatus Saccharibacteria’ was proposed as the new phylum name for TM7 based on the genomic analysis, which suggested these bacteria, with reduced genomes, consume sugar compounds primarily. However, the phylum was later designated as class *Ca*. Saccharimonadia within the phylum *Ca*. Patescibacteriota [24], and this phylum has now recently been validly published with the name *Minisyncoccota* [1].

The *Ca. N. lyticus* type strain TM7x^T^ cultivation breakthrough has formed the basis of extensive research into the physiology, growth and epibiont-host dynamics of phylum *Minisyncoccota* with a large body of literature [25-29]. The name *Ca. N. lyticus* type strain TM7x^T^ was first used in a publication in 2018 [30] and the etymology proposed in a preprint the same year [31], which was later published in 2020 [14]. The genus and species name *Ca. N. lyticus* was added to the List of Prokaryotic names with Standing in Nomenclature, citing the [14] paper [14], and is on the *International Journal of Systematic and Evolutionary Microbiology (IJSEM)* Candidatus List No. 3 of names of prokaryotic *Candidatus* taxa published in 2022 [32]. To provide taxonomic consistency for this important isolate, we propose the name *N. lyticus* (gen. nov. sp. nov.). We further propose that the associated family, order and class be *Nanosynbacteraceae* fam. nov., *Nanosynbacterales* ord. nov. and *Nanosynbacteria* class. nov.

## METHODS

### Isolation and cultivation

Saliva was collected from periodontally healthy subjects under the University of California, Los Angeles, Institutional Review Board (UCLA-IRB) no. 09-08-068-02A. Large debris and eukaryotic cells were removed by centrifuging saliva samples at 2,600 ***g*** for 10 min. Five hundred microlitres of the supernatant was inoculated into 5 ml of SHI medium [33] supplemented with 100 μg ml^−1^ streptomycin. This novel culture medium (SHI medium) supported the growth of many uncultivated oral bacteria within a multispecies community, including several uncultivated phylotypes of proposed class *Ca*. Nanosynbacteria (synonym *Ca*. Saccharibacteria) [33, 34]. Cultures were incubated at 37 °C under anaerobic conditions (85% N_2_, 5% CO_2_ and 10% H_2_). Samples were subcultured every 24 h for 5 days. Five hundred microlitres of culture was inoculated into 5 ml of fresh SHI medium starting at 100 μg ml^−1^ streptomycin and increasing by 100 μg ml^−1^ each day, resulting in 500 μg ml^−1^ streptomycin at day 5.

Daily cultures were sampled for these phylotypes by PCR. Five hundred microlitres of culture sample was used for DNA isolation using a MasterPure DNA Purification Kit (EPICENTRE). A specific primer set, TM7-580F (5′-AYTGGGCGTAAAGAGTTGC-3′) and TM7-1177R (5′-GACCTGACATCATCCCCTCCTTCC-3′) [4] was used to amplify a ~600 bp internal fragment of the 16S ribosomal RNA gene. PCR reactions contained 100 ng of purified genomic DNA, 40 pmol of each primer, 200 μM each dNTP, 4.0 mM MgCl2, 5 μl of 10× PCR buffer and 2.5 units of Taq DNA polymerase (Invitrogen) to a final volume of 50 μl. Cycling conditions were 96 °C for 3 min, followed by 35 cycles at 94 °C for 1 min, 64 °C for 1 min and 72 °C for 1 min, with a final extension period of 5 min at 72 °C. PCR products were evaluated by 1% agarose gel electrophoresis.

The phylotype enriched culture was then serially diluted in fresh SHI medium and plated onto SHI agar and incubated anaerobically at 37 °C until visible individual colonies formed. Colonies with distinct morphology were selected, inoculated into 5 ml of SHI medium supplemented with streptomycin (300 μg ml^−1^) and incubated at 37 °C under anaerobic conditions until turbid. Five hundred microlitres of each culture was used for DNA isolation using a MasterPure DNA Purification Kit, and the rest of the culture was frozen. For identification of bacterial isolates, the universal bacterial 16S rRNA primer pair 27F (forward: 5′-AGA GTT TGA TCM TGG CTC AG-3′) and 1492R (reverse: 5′-TAC GGY TAC C TT GTT ACG ACT T-3′) was used to generate a ~1,500-bp amplicon. PCR reactions contained 20 ng of genomic DNA, 200 μM each dNTP, 4.0 mM MgCl_2_, 100 nM each primer, 5 μl of 10× PCR buffer and 2.5 units of Taq polymerase to a final volume of 50 μl. Cycling conditions were 3 min at 94 °C for initial denaturation; 27 cycles at 94 °C for 1 min, 50 °C for 1 min and 72 °C for 2 min; and a final chain elongation at 72 °C for 5 min. The 16S rRNA gene 1,500 bp amplicons were purified using a QIAquick PCR Purification Kit (QIAGEN) and sequenced at the UCLA sequencing and genotyping core facility. Sequences were searched using blast against the Human Oral Microbiome Database (https://www.homd.org/) and National Center for Biotechnology Information (https://www.ncbi.nlm.nih.gov/) databases, with 98–100% identity to deposited sequences in the public domain databases considered to be positive identification of taxa.

A subset of colonies contained a mixture of a specific phylotype (TM7x^T^) and host bacteria *Pauljensenia odontolytica_C* strain XH001. No colonies of pure TM7x^T^ were detected. However, colonies of pure XH001 were identified. Colonies containing TM7x^T^ and XH001 were cultured in brain heart infusion (BHI) medium at 37 °C under microaerophilic conditions (2% O_2_, 5% CO_2_ and 93% N_2_) using a Whitley anaerobic workstation. The exponential phase planktonic cocultures were collected, and frozen stocks were made and stored at −80 °C.

### Growth

Cocultures were grown in either Bacto BHI (BD) medium or SHI medium at 37 °C. Cultures were grown either anaerobically (85% N_2_, 5% CO_2_ and 10% H_2_) or under microaerophilic conditions (2% O_2_, 5% CO_2_ and 93% N_2_).

### Monitoring growth of the cocultures

Host bacteria cell growth was monitored by optical density (OD600) and c.f.u. [35]. Due to the small size of TM7x^T^, the epibiont was detected using phase-contrast imaging, quantitative polymerase chain reaction (qPCR) and the percentage of irregular host cell colonies [35]. Microscopy was used to assess TM7x^T^ presence on host bacteria cells and free-floating within the cultures. qPCR reactions were run with XH001 or TM7x^T^ specific primers yielding ratios between the species. Host cell colonies showed differential morphology based on the presence of TM7x^T^, with the epibiont inducing irregular morphology. The percentage of irregular colonies indicated the percentage of host cells in each coculture with attached TM7x^T^.

### Infection assays

For the original infection assay [18], cells were separated from their host by repeatedly passing the coculture through a 28-gauge needle and filtering through a 0.22-μm filter. Isolated cells were mixed with potential host cells and incubated anaerobically for 72 h, periodically monitored under microscopy. For host range assays [35, 36], TM7x^T^ cells were filtered through 0.45-μm polyvinylidene fluoride membranes and subjected to ultracentrifugation at 120,000 ***g*** at 4 °C for 90 min. Isolated free-floating TM7x^T^ were mixed with potential host cells, and the resultant cocultures were passaged every 24 to 48 h for at least five passages. The presence of TM7x^T^ cells in the final cultures was confirmed by microscopic examination and specific polymerase chain reaction (see the ‘Isolation and cultivation’ section).

### Microscopy

Phase contrast images were acquired during growth in the indicated cultures. Cells were directly observed and imaged using a Nikon Eclipse E400 microscope equipped with a Nikon Plan Fluor 100×/1.30 oil immersion objective.

Fluorescence *in situ* hybridization (FISH) was performed based on a previously published protocol [37]. Briefly, cells were fixed in 4% formaldehyde for 3 h, followed by enzymatic permeabilization using 2 mg ml^−1^ lysozyme prepared in 20 mM Tris buffer (pH 7.0) for 9 min at 37 °C. After fixation, cells were resuspended in 500 μl hybridization buffer containing 20 mM Tris-HCl (pH 8.0), 0.9 M NaCl, 0.01% SDS and 30% deionized formamide and pre-incubated at 37 °C for 30 min. Hybridization was carried out for 3 h at 42 °C using a Cy5-labelled TM7x^T^-specific probe (TM7-567:5′-CCTACGCAACTCTTTACGCC-3′) and a Cy3-labelled XH001-specific probe (M33910:5′-CAGTGTCGCCGTGCAT-3′). For visualization, Cy5-labelled TM7x^T^ signals were displayed in white to enhance contrast. Following hybridization, samples were washed three times in 0.1×saline–sodium citrate buffer for 15 min per wash and mounted using SlowFade Gold antifade reagent (Invitrogen, S36937). Imaging was performed on a Leica SPE I inverted confocal microscope using an ACS APO 100×/1.15 oil-immersion objective. Each experiment was repeated independently.

For scanning electron microscopy imaging, poly-l-lysine-treated 12-mm round glass coverslips were placed into 24-well plates, each well containing 1 ml of medium. Either 100 μl of 24 h XH001 monocultures or cocultures consisting of XH001 and TM7x^T^ were added to each well and incubated for 24 h. Coverslips were then retrieved and fixed overnight in a mixture of 4% glutaraldehyde, 3% paraformaldehyde and 0.05% ruthenium red prepared in 0.1 M cacodylate buffer. Following fixation, samples were rinsed three times for 10 min each in 0.1 M cacodylate buffer and subsequently post-fixed for 1 h in 1% osmium tetroxide supplemented with 0.05% ruthenium red in the same buffer. Coverslips were washed twice in distilled water for 10 min per wash and dehydrated through a graded ethanol series (50%, 70% and 80% for 10 min each), followed by three sequential incubations in 95% ethanol for 10 min each. Samples were then critical-point dried to preserve cellular morphology and sputter-coated with a 5-nm layer of platinum–palladium. Imaging was performed using a field-emission scanning electron microscope (Zeiss Ultra 55 FE-SEM) operated at 2 kV, with a working distance of 3–5 mm, using either secondary electron or in-lens detectors.

### Whole-genome phylogenetic analysis

The complete closed circular genome sequence (CP007496.1, RefSeq GCF_000803625.1) was published previously [18]. Taxonomic assignment using the *Ca. N. lyticus* strain TM7x^T^ whole genome was derived from the analysis and provided in the Genome Taxonomy Database (GTDB) (https://gtdb.ecogenomic.org/) [24, 38-40]. The bac120.tree based on the r220 bac120 markers was downloaded from GTDB, loaded into Archaeopteryx (https://www.phylosoft.org/archaeopteryx/) and used to generate a subtree containing GTDB *Ca*. Patescibacteriota (the GTDB version r220 synonym for the validly published *Minisyncoccota*). The subtree was imported into the Interactive Tree of Life (https://itol.embl.de/) to display the tree at different levels of taxonomy. Average nucleotide identity was calculated between TM7x^T^ and the other species in the GTDB *Ca. Nanosynbacter* genus using skani [41].

### 16S rRNA gene tree

The 16S rRNA sequence of *N. lyticus* strain TM7x^T^ derived from the complete genome was also deposited (OM390184.1) [18]. For the 16S rRNA gene-based tree construction, we used nearly complete or full-length 16S rRNA gene sequences derived from predominantly complete genomes collected for the phyla *Minisyncoccota* and the phyla *Chloroflexota, Actinomycetota, Cyanobacteriota* and *Bacillota*. A total of 108 sequences were selected from representative strains across these phyla. Selected sequences were aligned using MAFFT v7.475 [42] using default parameters, and a maximum-likelihood tree using RAxML version 8.2.12 [43] with a GTR-GAMMA model was generated.

### Deposition

Strain TM7x^T^ has been deposited in the ATCC (ATCC TSD-290^T^, deposition August 2023 https://www.atcc.org/products/tsd-290) collection as a coculture with its host *Pauljensenia odontolytica_C* strain XH001 (ATCC BAA-3209).

## RESULTS AND DISCUSSION

### Sample description, habitat and isolation

*N. lyticus* type strain TM7x^T^ was first isolated on 12 May 2013 as described by He *et al*. [18]. Saliva samples collected from periodontally healthy subjects were subjected to serial enrichment on SHI media with streptomycin selection (see the ‘Methods’ section). SHI medium was developed to support the growth of uncultivated oral bacteria within a multispecies community, including several phylotypes of class *Ca*. Nanosynbacteria (synonym *Ca*. Saccharibacteria) [33, 34]. The presence in enriched samples was detected using class-specific PCR primers [18]. Enriched samples were then plated for single colonies, and the resulting isolates were identified by universal bacterial 16S rRNA primer PCR sequencing. Sequences with 98–100% identity to those sequences deposited in the public domain databases were considered to be positive identification of taxa. A subset of the isolates contained two previously uncultivated species, *Nanosybacter lyticus* TM7x^T^ and *Pauljensenia odontolytica_C* strain XH001. While pure XH001 colonies were found, TM7x^T^ only appeared with XH001, suggesting a symbiotic relationship between the species. Physical and chemical means were used to disrupt the attachment to isolate pure cultures of XH001 and TM7x^T^. However, no TM7x^T^ colonies were obtained. Addition of spent coculture medium or heat-killed XH001 also did not yield independent growth of TM7x^T^ [18], without the host bacteria present.

### Growth and physiology

Microscopic examination of independently isolated TM7x^T^ and XH001 cocultures showed TM7x^T^ to be small (200–300 nm), rod-shaped cells with a monoderm-like cell wall physically bound to the XH001 cells [18] (Fig. 1a). Microscopy also revealed that cocultures were a mixture of cells, including infected host, uninfected host and free-floating independent TM7x^T^ [35, 37] (Fig. 1b). TM7x^T^-associated XH001 cells showed elongated and branched morphology [37] (Fig. 1c). While isolated under anaerobic conditions with SHI media, the cocultures were found to grow in BHI media anaerobically, with oxygen and carbon dioxide (19.7% O_2_, 5% CO_2_), or under microaerophilic conditions (2.6% O_2_, 5% CO_2_), but not under atmospheric conditions (20.9% O_2_, 0% CO_2_) [37]. Of these, the growth of XH001 was optimal under microaerophilic conditions. The presence of TM7x^T^ was also found to slow the overall growth of XH001 in the coculture. XH001 alone had an estimated generation time of 120±18 min under microaerobic conditions, but 153±8 min for the coculture [36].

The episymbiotic lifestyle of TM7x^T^ was confirmed by establishing cocultures using isolated TM7x^T^ [18]. TM7x^T^ was isolated from its host bacteria by repeatedly passing the coculture through a 28-gauge needle and filtering the mixture through a 0.22-μm filter. XH001 and isolated TM7x^T^ cells were mixed and incubated anaerobically at 37 °C, resulting in new cocultures of TM7x^T^ attached to the host. Establishing new cocultures also reveals a complex interplay between epibiont and host bacteria [35]. Naïve XH001, a host that had never been exposed to TM7x^T^, showed a multi-stage adaptation when mixed at a 1:3 ratio with TM7x^T^. Both XH001 and TM7x^T^ populations were monitored. Because it is difficult to detect TM7x^T^ by traditional bacterial methods, a modified viral counting procedure was employed [35] to quantify TM7x^T^ cells. Meanwhile, microscopy, qPCR and the percentage of host colonies showing an irregular morphology were used to determine the percentage of host cells carrying TM7x^T^. During the initial infection phase, the overall host growth rate was mostly unaffected while the TM7x^T^ population increased. As the TM7x^T^ population reached its maximum, there was a killing phase where host cell numbers dropped dramatically. During this time, qPCR estimated very high epibiont loads, >50 TM7x^T^ per host cell [35]. The crash was followed by a recovery phase where host cell levels recovered to those seen with uninfected XH001, while TM7x^T^ showed reduced loads per host and lower percentage irregular colony morphology. The cocultures eventually reached a stable symbiosis between epibiont and host bacteria. When subcultured into new media, the stable cocultures did not show the large fall off in host numbers but grew together as a stable coculture. Because XH001 cells physically interacting with TM7x^T^ showed virtually no growth, but cocultures include a non-infected subpopulation of XH001, it was theorized that the dividing non-infected XH001 provide new host cells for horizontal transfer of TM7x^T^.

The ability to establish new cocultures was used to examine host range [44]. Attempts were made to establish TM7x^T^ on several *Actinomyces* strains, close relatives to *Pauljensenia. odontolyticus*. The only amenable hosts were from the subclade *Pauljensenia* (*Pauljensenia odontolytica_C* strain XH001, *Pauljensenia sp000185285* strain F0310 and *Pauljensenia meyeri* strain W712). Other isolated *Ca*. Nanosynbacteria (synonym *Ca*. Saccharibacteria) species showed different host ranges and were unable to establish an episymbiotic relationship with the *Pauljensenia* strains, indicating that host range was relatively restricted and species specific [36].

While TM7x^T^ was shown to be an obligate epibiont of its host, the specifics of this interaction are still an area of active research. Pili appear to play a role in host association [28, 29, 45, 46]. TM7x^T^ expresses two distinct types of IV pili. The thin pili (1.8 nm diameter) were found to be non-essential, but deletion of pili components reduced host binding. The thick type IV pili (3.2 nm diameter) was determined to be essential and provide twitch motility [45, 46]. Presumably, motility is important for bringing free-floating TM7x^T^ into contact with new host cells.

In terms of metabolism, the nutrient transfer method from the host remains unknown. Genome analysis indicated glycolysis as the primary energy pathway [25]. However, in contrast to environmental members, mammalian-associated *Ca*. Nanosynbacteria (synonym *Ca*. Saccharibacteria), including TM7x^T^, have acquired a complete arginine deaminase locus [14, 27]. This system allows the import and catabolism of arginine for ATP production in TM7x^T^ [27]. How important arginine is to attached TM7x^T^ is unclear, but the presence of arginine supplementation maintained survival in the absence of their host bacteria [27]. Cells with arginine also maintained higher infectivity when transferred to new host bacteria than those incubated in the absence of arginine.

### Genomic sequencing

The complete, closed, circular genome of TM7x^T^ has been sequenced (Bioproject PRJNA241438, Genbank CP007496.1, GenBank assembly GCA_000803625.1, Refseq NZ_CP007496.1, RefSeq assembly GCF_000803625) [18]. The genome is 705,138 nt long with a 44.5 mol% G+C content. RefSeq annotation revealed 754 predicted genes, 701 protein-coding, single copies of 5S, 16S, 23S rRNA and 44 predicted tRNA [47]. As with other members of the phylum, genes for many essential biosynthesis pathways, such as amino acids, vitamins and cell wall precursors, are absent [2, 14, 48].

### Phylogenetic analysis

16S rRNA gene-based tree phylogenetic analyses have been published previously [14, 15, 18, 25, 31, 36]. In this repeated analysis, we included nearly complete or full-length 16S rRNA gene sequences derived from predominantly complete genomes collected for phylum *Minisyncoccota*, including the class *Ca*. Nanosynbacteria, the class *Ca*. Microgenomatia and the phyla *Chloroflexota, Actinomycetota, Cyanobacteriota* and *Bacillota* (Fig. 2). The tree showed placement of *N. lyticus* strain TM7x^T^ with the other *Ca*. Nanosynbacter species.

Phylogenomic analysis was assessed using the GTDB tree based on the r220 bac120 marker genes (Fig. 3) (https://gtdb.ecogenomic.org/) [24, 38-40]. TM7x^T^ falls under the phylum *Minisyncoccota* (synonym *Ca*. Patescibaceriota) [1]. In GTDB, the phylum contains thirty class-level domains; however, only 11 (c_Dojkabacteria, c_WWE3, c_4484–211, c_Microgenomatia, c_UBA1384, c_CPR2, c_Gracilibacteria, c_JAEDAM01, c_Paceibacteria, c__ABY1 and the proposed c_Nanosynbacteria) contain sufficient species to have multiple orders, with several classes represented by only a single species. Most classes contain solely environmental species. Only three, the proposed class *Ca*. Nanosynbacteria, as well as *Ca*. Absconditabacteria (synonym SR-1) and *Ca*. Gracilibacteria (synonym GN02), contain members associated with mammalian microbiomes [14, 18, 31].

Within the GTDB taxonomic tree, the proposed order *Ca*. Nanosynbacterales comprises the vast majority of species in class *Ca*. Nanosynbacteria, 2,277 out of 2,355. The biogeography of this order is broad, and members have been detected in environmental samples from terrestrial, freshwater and marine sources as well as activated sludge bioreactors (reviewed in [13, 14, 49]). In addition, many members have been observed to be mammalian host-associated. While the families that make up this order in the GTDB have been observed primarily through large-scale sequencing projects, we provided the first genomic descriptions for several of the mammalian-associated family members [14]. Two members of the family *Ca*. Nanosyncoccaceae (synonym G3) were derived from the moose rumen and the human oral cavity with G+C content of 43% and 40%, respectively. Two members of *Ca*. Nanoperiomorbaceae (synonym G5) came from the human oral cavity and possessed ~50% GC content. Finally, a member of *Ca*. Nanogingivalaceae (synonym G6) was also derived from the human oral cavity but showed a much lower G+C content of 32%. These lineages are present at high prevalence within hosts [14]. Analyses suggest that multiple acquisition events in the past led to the current wide diversity, with convergent evolution of key functions allowing *Ca*. Nanosynbacteria (synonym *Ca*. Saccharibacteria) from the environment to adapt to mammals. A model of temporally separated acquisition events was proposed to explain the observed diversity of *Ca*. Nanosynbacteria (synonym *Ca*. Saccharibacteria) within mammals [14].

GTDB shows the family *Ca*. Nanosynbacteraceae currently contains only one genus, the candidate genus *Nanosynbacter*. This family contains almost all of the isolated and stably cultivated species within the order [16, 18, 36, 50, 51]. As mentioned, most families within the order *Ca*. Nanosynbacterales contain environmental species. However, *Ca*. Nanosynbacteraceae are predominantly associated with mammalian microbiomes [14, 31]. This trait is not exclusive to *Ca*. Nanosynbacteraceae; however, *Ca*. Nanogingivalaceae, *Ca*. Nanoperiomorbaceae and *Ca*. Nanosyncoccaceae are also predominantly mammalian-associated [14, 31]. Distinctions between *Ca*. Nanosynbacteraceae and the other families in order *Ca*. Nanosynbacterales are seen in the G+C content and across the genomes. A shared set of genes, anaerobic ribonucleoside-triphosphate reductase (*nrd*D) and lactate dehydrogenase (*ldh*), is found in human associated strains but is not present in environmental strains. They also show distinct lineages between the mammalian-associated families [14]. The *Ca*. Nanosynbacteraceae strains with *nrd*D show a gene that is phylogenetically clustered to the *Firmicutes* phyla from the class *Clostridia*, while *nrd*D from the other mammalian-associated lineages clustered with class *Bacilli* or a group of *Proteobacteria* and *Elusimicrobia*. Similarly, *ldh* had indications of disparate horizontal transfer into the different families. *Ca*. Nanosynbacteraceae *ldh* specifically clustered with the *Bacillota*.

The *Ca*. Nanosynbacteraceae do show significantly high genomic synteny with members of some other families [14]. This includes *Saccharimonas aalborgensis* in *Ca*. Saccharimondadaceae and UBA1020 sp000503915, synonym RAAC3, in *Ca. UBA1020*. Intriguingly, these are distinctly environmental groups in contrast to the mammalian-associated *Ca*. Nanosynbacteraceae, indicating that the members across these families have not rearranged their genomes drastically during acquisition and adaptation within mammalian hosts, which suggests more recent acquisition in mammals than the other families, as was previously proposed [14].

Candidate genus Nanosynbacter is the only genus within *Ca*. Nanosynbacteraceae. Candidate species *Nanosynbacter lyticus* strain TM7x^T^ is the type strain of the genus. *Ca*. Nanosynbacter species range in G+C content from 42–53%. Members have also shown high levels of chromosomal synteny with closely related species [14]. Average nucleotide identity (ANI) was determined between TM7x^T^ and the other members of this genus in GTDB using skani [41]. ANI ranged from 76 to 93% with an average of 82±2%, consistent with a distinct species.

### Note

Rule 30 (3b) of the International Code of Nomenclature of Prokaryotes states that the valid publication of a species name necessitates deposition in at least two publicly accessible culture collections in different countries [52]. A committee consisting of the Chair of the International Committee on Systematics of Prokaryotes (ICSP), the Chair of the Judicial Commission of the ICSP and the Editor-in-Chief of the *IJSEM* has granted an exception to ICNP Rule 30(3)(b) regarding deposition of the obligate epibiont *N. lyticus* strain TM7x^T^. Strain TM7x^T^ has been deposited in the ATCC (ATCC deposition August 2023 https://www.atcc.org/products/tsd-290, accession TSD-290^T^) collection as a coculture with its host bacterium *Pauljensenia odontolytica_C* strain XH001. Efforts are currently underway to deposit Strain TM7x^T^ in a recognized culture collection in another country.

## DESCRIPTION OF THE *NANOSYNBACTERIA* CLASS. NOV.

(Na.no.syn.bac.te’ri.a. N.L. masc. n. *Nanosynbacter*, a bacterial genus name. -*ia*, ending to denote a class; N.L. neut. pl. n. *Nanosynbacteria*, the Nanosynbacter class)

The class belongs to the phylum *Minisyncoccota*. The description of the class *Nanosynbacteria* is the same as that of the genus *Nanosynbacter*. The type genus is *Nanosynbacter*.

## DESCRIPTION OF THE *NANOSYNBACTERALES* ORD. NOV.

(Na.no.syn.bac.te.ra’les. N.L. masc. n. *Nanosynbacter*, a bacterial genus name. -*ales*, ending to denote an order; N.L. fem. pl. n. *Nanosynbacterales*)

The order belongs to the class *Nanosynbacteria* of the phylum *Minisyncoccota*. The description of the order *Nanosynbacterales* is the same as that of the genus *Nanosynbacter*. The type genus is *Nanosynbacter*.

## DESCRIPTION OF THE *NANOSYNBACTERACEAE* FAM. NOV.

(Na.no.syn.bac.te.ra.ce’ae. N.L. masc. n. *Nanosynbacter*, a bacterial genus name. -*aceae*, ending to denote a family; N.L. fem. pl. n. *Nanosynbacteraceae*)

The family belongs to the order *Nanosynbacterales* of the class *Nanosynbacteria*. The description of the family *Nanosynbacteraceae* is the same as that of the genus *Nanosynbacter*. The type genus is *Nanosynbacter*.

## DESCRIPTION OF THE *NANOSYNBACTER* GEN. NOV.

(Na.no.syn.bac’ter. Gr. masc. n. *nanos*, a dwarf; Gr. prep. *syn*, together; N.L. masc. n. *bacter*, a rod; N.L. masc. n. *Nanosynbacter*, a dwarf symbiotic rod)

The genus belongs to the family *Nanosynbacteraceae* of the order *Nanosynbacterales*. The species type is *N. lyticus*.

## DESCRIPTION OF *NANOSYNBACTER LYTICUS* SP. NOV.

(ly’ti.cus. N.L. masc. adj. *lyticus* (from Gr. masc. adj. *lytikos*), able to dissolve, dissolving)

Cells are small (200–300 nm) and rod-shaped with a monoderm-like cell wall. They are found free-floating or attached to host bacteria, *Pauljensenia* cells. They are obligate epibionts that grow under anaerobic or microaerophilic conditions with their host bacteria. Genome sequencing determined a G+C content of 44.5%, and no other genomes had sufficient ANI scores (>95%) to be considered affiliated with this species. The type strain *N. lyticus* strain TM7x^T^ was obtained from enrichment cultures derived from healthy human saliva obtained at the University of California, Los Angeles, Los Angeles, USA. The species belongs to the genus *Nanosynbacter* of the family *Nanosynbacteraceae*. The complete genome and 16S rRNA gene sequence for *N. lyticus* strain TM7x^T^ were deposited with Genbank under accession numbers CP007496.1, RefSeq GCF_000803625 and OM390184.1, respectively. The ATCC accession number for the strain is TSD-290^™^.

## Figures and Tables

**Fig. 1.**
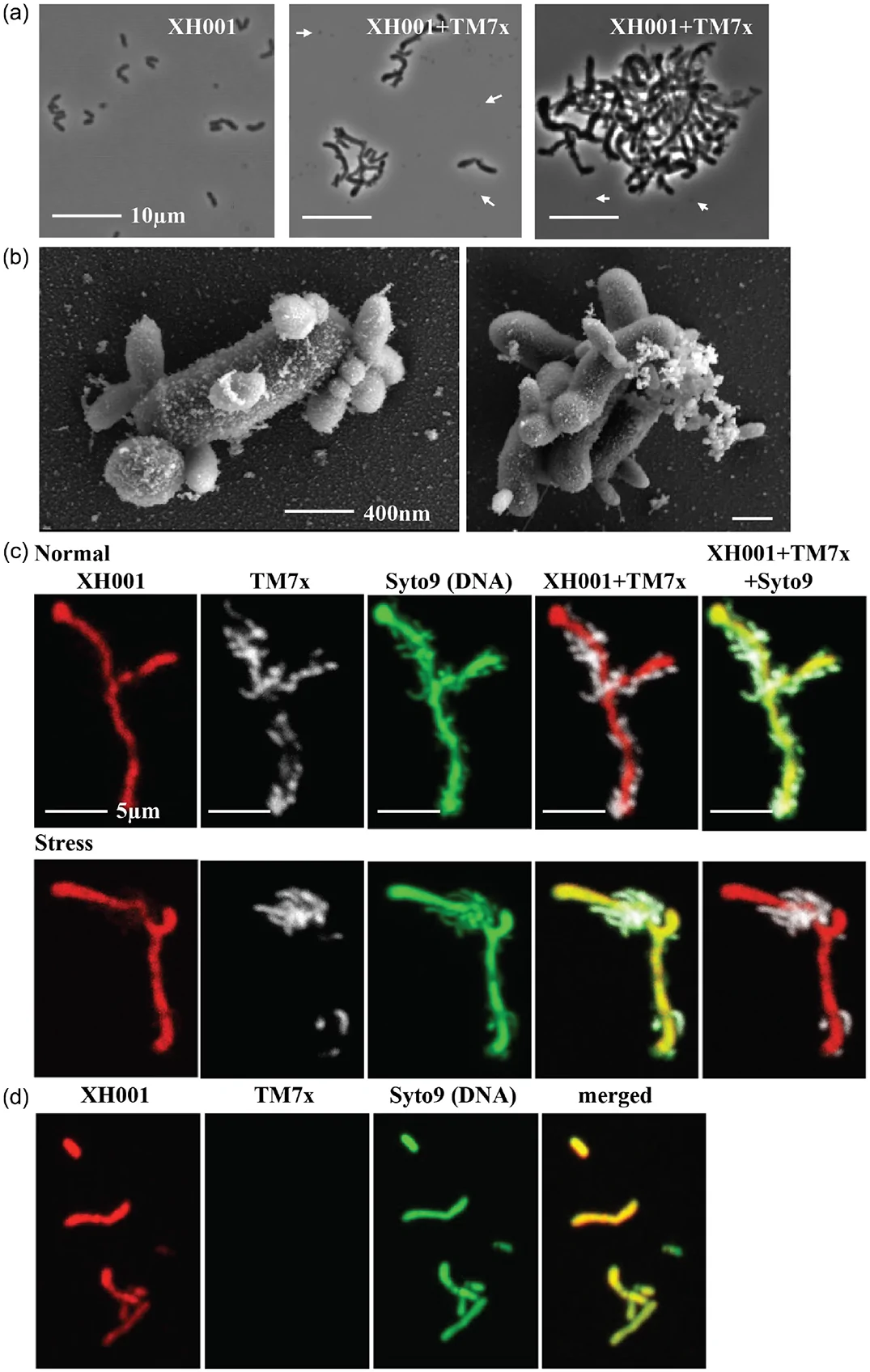
*N. lyticus* attached to *Pauljensenia odontolytica_C*. (a) Phase contrast images of XH001 alone and with TM7xT. Shows TM7xT decorating the surface of the host bacteria. Where TM7xT induces stress in XH001, conditions, such as host killing in growth crash, low oxygen or during overgrowth of TM7xT, radiating colonies can form from clumping together of the elongated hyphal XH001 cells. TM7xT can detach from the host bacteria to form free-floating TM7x (see arrows), suggesting potential sources of horizontal infection. (b) Scanning electron microscopy images show attached small TM7xT bacteria on the surface of the host bacteria. TM7xT forms a bowling pin-like structure and appears to grow by budding. (c) FISH imaging of TM7xT and its host bacteria XH001. DNA probes specific to TM7xT or XH001 were used to separately stain and visualize the two bacteria. Universal syto9 stained the genomic DNA of both bacteria. (d) FISH staining of XH001 is shown as a control.

**Fig. 2.**
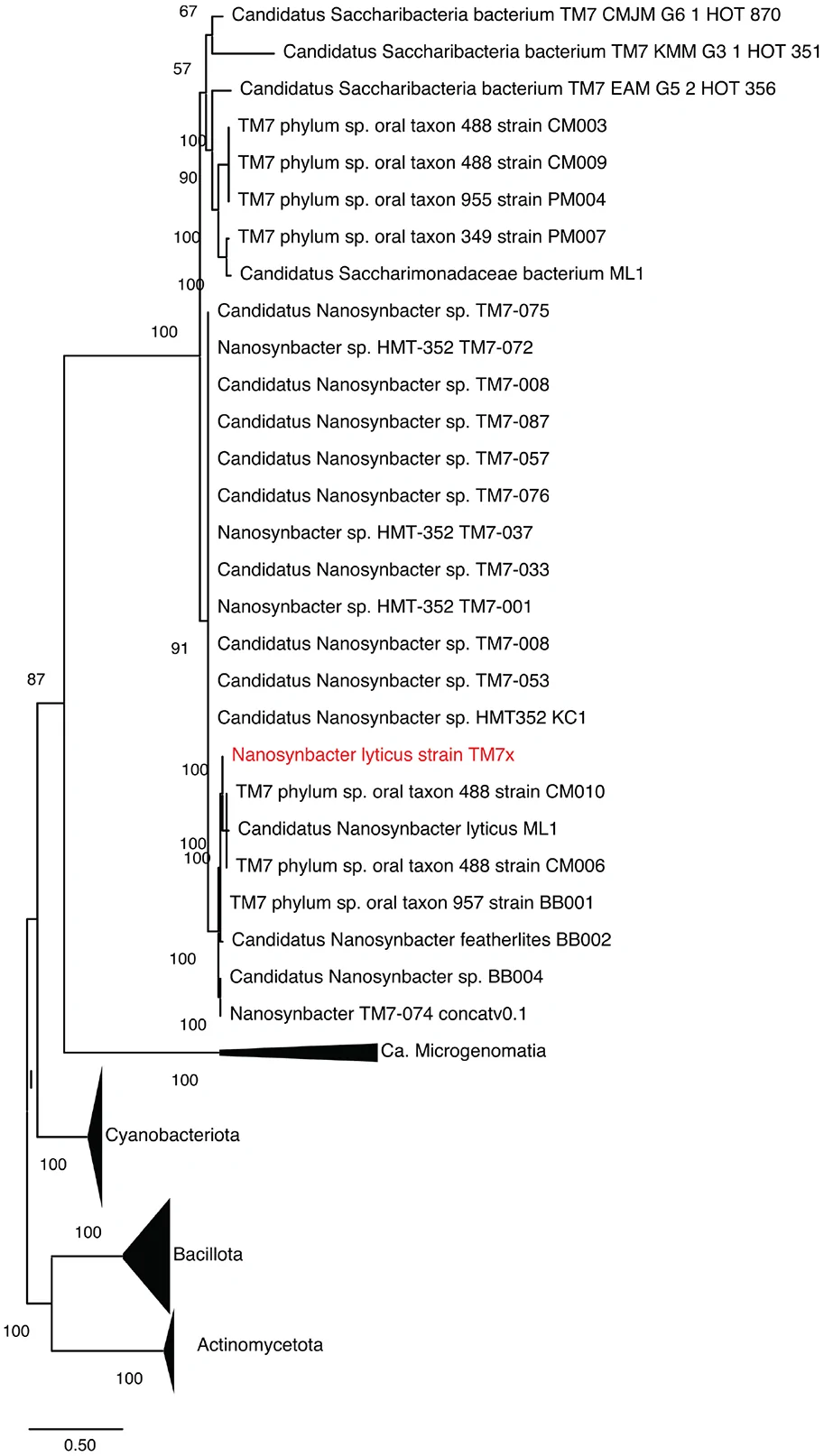
Maximum-likelihood tree based on 16S rRNA gene sequences. Representative sequences across class Nanosynbacteria and other Bacteria phyla derived from near or full-length 16S rRNA genes in predominantly complete bacterial genomes, where available, were used to construct the tree. Bootstrap values are shown. *N. lyticus* strain TM7xT is indicated in red.

**Fig. 3.**
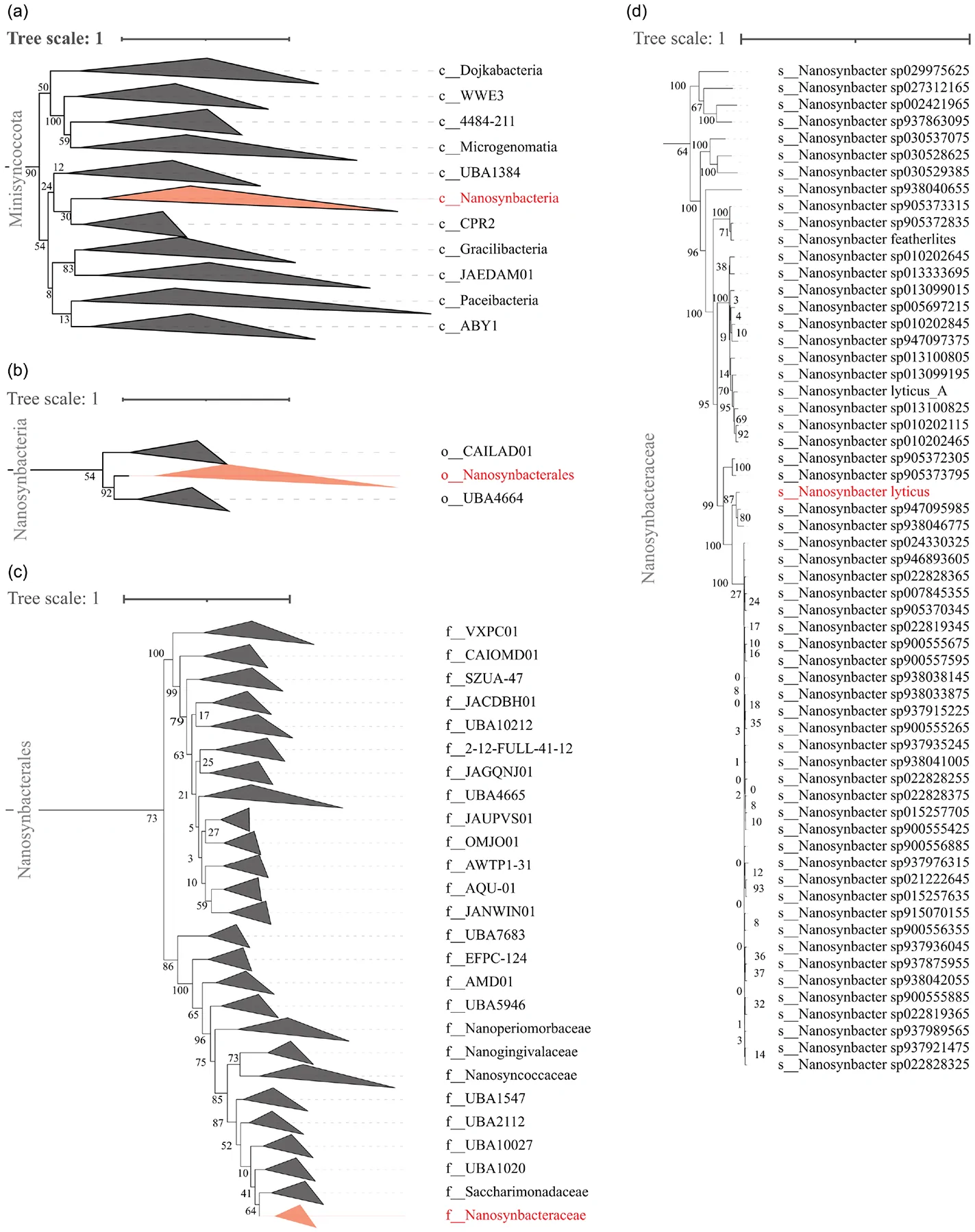
Taxonomic tree of *N. lyticus*. A tree based on the r220 bac120 markers from GTDB at different taxonomic levels. (a) Classes of phylum *Minisyncoccota*. (b) Orders of class Nanosynbacteria. (c) Families of order Nanosynbacterales. (d) Species of the family Nanosynbacteracea. The family contains only the genus Nanosynbacter. Red indicates groups with proposed nomenclature. Numbers indicate bootstrap values. For clarity, only clades that include Nanosynbacter or cover multiple clades of the next taxonomic level are shown.

## Abbreviations:

ANI: average nucleotide identity
BHI: brain heart infusion
CPR: candidate phyla radiation
FISH: fluorescence *in situ* hybridization
GTDB: Genome Taxonomy Database
ICSP: International Committee on Systematics of Prokaryotes
*IJSEM*: *International Journal of Systematic and Evolutionary Microbiology*
